# Nature documentaries vs. quiet rest: no evidence for an impact on event-related desynchronization during motor imagery and neurofeedback

**DOI:** 10.3389/fnhum.2025.1539172

**Published:** 2025-04-08

**Authors:** Jennifer Decker, Mareike Daeglau, Catharina Zich, Cornelia Kranczioch

**Affiliations:** ^1^Neurocognition and Functional Neurorehabilitation Group, Neuropsychology Lab, Department of Psychology, Carl von Ossietzky University Oldenburg, Oldenburg, Germany; ^2^Cluster of Excellence “Hearing4all”, Carl von Ossietzky University Oldenburg, Oldenburg, Germany; ^3^Department of Clinical and Movement Neurosciences, UCL Queen Square Institute of Neurology, Oxford, United Kingdom; ^4^Nuffield Department of Clinical Neurosciences, Wellcome Centre for Integrative Neuroimaging, FMRIB, University of Oxford, Oxford, United Kingdom; ^5^Research Center Neurosensory Science, Carl von Ossietzky University Oldenburg, Oldenburg, Germany

**Keywords:** motor imagery, neurofeedback, EEG, event-related desynchronization, nature documentaries, quiet rest, context factor, BCI

## Abstract

Motor imagery (MI) in combination with neurofeedback (NF) has emerged as a promising approach in motor neurorehabilitation, facilitating brain activity modulation and promoting motor learning. Although MI-NF has been demonstrated to enhance motor performance and cortical plasticity, its efficacy varies considerably across individuals. Various context factors have been identified as influencing neurophysiological outcomes in motor execution and MI, however, their specific impact on event-related desynchronization (ERD), a key neurophysiological marker in NF, remains insufficiently understood. Previous research suggested that declarative interference following MI-NF may serve as a context factor hindering the progression of ERD. Yet, no significant changes in ERD within the mu and beta (8–30 Hz) frequency bands were observed across blocks in either a declarative interference or a control condition. This raises the question of whether the absence of ERD modulation could be attributed to the break task that was common to both declarative interference and control condition: watching nature documentaries immediately after MI blocks. To investigate this, we conducted a follow-up study replicating the original methodology while collecting new data. We compared NF-MI-ERD between groups with and without nature documentaries as a post-MI condition. Participants completed three sessions of kinesthetic MI-NF training involving a finger-tapping task over two consecutive days, with quiet rest as the post-MI condition (group *quiet rest*). 64-channel EEG data were analyzed from 17 healthy participants (8 females, 18–35 years, M and SD: 25.2 ± 4.2 years). Data were compared to a previously recorded dataset (group *documentaries*), in which 17 participants (10 females, 23–32 years, M and SD: 25.8 ± 2.5 years) watched nature documentaries after MI blocks. The results showed no significant main effects for blocks or group, though a session-by-group interaction was observed. Post-hoc tests, however, did not reveal significant differences in ERD development between the groups across individual blocks. These findings do not provide evidence that nature documentaries used as a post-MI condition negatively affect across-block development of NF-MI-ERD. This study highlights the importance of exploring additional context factors in MI-NF training to better understand their influence on ERD development.

## 1 Introduction

Motor imagery (MI) is the mental simulation of movements without actual muscle activity (Ruffino et al., 2017; Wajda et al., 2021) allowing for repetitive practice of motor tasks, even in the absence of residual movement. According to the theory of neural simulation of action (Jeannerod, 2001), MI involves neural substrates that are also active during motor execution, thus making them functionally comparable (Gentili et al., 2010; Williams et al., 2011; Hetu et al., 2013). This overlap makes MI particularly valuable in neurorehabilitation, where traditional physical exercises may be limited. Therefore, it represents a promising treatment method for supporting functional motor recovery, particularly in the neurorehabilitation of patients with motor impairments such as stroke (Zich et al., 2017; Villa-Berges et al., 2023). MI can be supplemented by real-time neurofeedback (NF) (Hwang et al., 2009; Gomez-Pilar et al., 2016), a technique that enables individuals to volitionally modulate their brain activity while receiving additional sensory input (Onagawa et al., 2023), thereby enhancing MI performance (Neuper et al., 2009; Ono et al., 2013). MI-NF is most frequently based on the event-related desynchronization (ERD), a decrease in sensory-motor power in the alpha and beta frequency ranges, reliably detectable when present by electroencephalography (EEG) (Pfurtscheller and Neuper, 2006; Morash et al., 2008; Jeon et al., 2011). Numerous studies have examined the effects of MI (Ladda et al., 2021) and NF (Onagawa et al., 2023) on motor performance. Studies show that MI-NF training improves motor function (Pichiorri et al., 2015) and lateralizes ERD (Zich et al., 2017) in stroke survivors. In healthy individuals, it enhances sensorimotor cortex activity (Wang et al., 2019) and white matter connectivity (Marins et al., 2019).

Despite growing interest in MI-NF, standardized protocols for optimizing its effectiveness remain lacking. Research has yet to establish clear guidelines regarding session structure, training protocols, and feedback mechanisms, leading to inconsistencies across studies (Vernon et al., 2009; Stefano Filho et al., 2024). Sensorimotor oscillations, particularly in the beta band, are shaped by multiple factors such as task complexity, cognitive demands, and attentional processes (Kilavik et al., 2013), all of which influence ERD development and contribute to variability in MI-NF outcomes. Beyond methodological inconsistencies, variability also arises from individual differences in ERD patterns, which have been shown to correlate with brain-computer interface performance (Rimbert et al., 2022) and fluctuate across different MI tasks within the same individual (Wriessnegger et al., 2020). Various context factors, such as the environment and individual cognitive states, likely influence motor skill learning, MI-NF effectiveness, and its usability outside of laboratory environments (Jeunet et al., 2015; Daeglau et al., 2021a). They presumably explain the inter-and intraindividual differences observed in study outcomes (Ahn and Jun, 2015), highlighting the need for further investigation to develop practical, innovative MI approaches (Ladda et al., 2021). These variations make it difficult to compare findings and assess the reliability of MI-NF interventions. Understanding how different context factors interact with brain processes in MI-NF training is essential for improving its reliability and effectiveness.

Daeglau et al. (2021b) examined how sleep and declarative interference as possible context factors affect MI-NF development across blocks. These factors were chosen based on prior findings in non-MI-NF studies, where declarative interference after MI practice negatively impacted subsequent motor performance (Debarnot et al., 2012), and sleep played a critical role in motor skill consolidation following ME (Brown and Robertson, 2007). Declarative interference refers to cognitive tasks that engage declarative memory processes, which can overlap with motor learning by competing for the same cognitive resources (Gagne and Cohen, 2016), such as working memory and attention. In the study by Daeglau et al. (2021b), three experimental groups underwent MI-NF training under different conditions: no interference (passively watching nature documentaries), immediate interference, and late interference. The interference consisted of four non-motor tasks designed to challenge declarative memory, including a word list recall, an n-back task, a face-name matching task, and a modified version of the Paced Auditory Serial Addition Test (PASAT) (Gronwall, 1977). While NF enhanced ERD within MI blocks, no differences were found between the groups, and the typical increase in ERD over MI blocks (Ono et al., 2013; McWhinney et al., 2018; Foldes et al., 2020) was absent. As a break and control block, a set of nature documentaries was chosen due to their specific characteristics: they included atmospheric music, featured no visible human interactions, and were narrated by a speaker. These elements were intended to minimize social and motor engagement while maintaining a mild cognitive load. However, in their discussion, Daeglau et al. (2021b) raised concerns that watching nature documentaries might not have served as an optimal control task, as they could have induced drowsiness or distraction, potentially affecting the results. Supporting this notion, Jacquet et al. (2021) found that both prolonged MI task and watching a documentary as a control condition increased feelings of fatigue. In their study, participants engaged in 50 minutes of MI and 50 minutes of watching a documentary, with both conditions resulting in a significant rise in fatigue from pre-to post-measurement. Although this was not a systematic investigation of fatigue induced by watching a documentary but rather an incidental finding, it is plausible that a similar fatigue effect occurred in Daeglau et al. (2021b), which may have influenced ERD. Documentaries are commonly used as a control condition in experimental designs assessing motor skills (Bassolino et al., 2014; Ruffino et al., 2019; Hilt et al., 2023), but their potential influence on ERD within the context of MI-NF training remains unclear. Given that ERD is sensitive to cognitive and attentional states, understanding the impact of watching nature documentaries immediately after performing MI-NF training on future ERD development provides valuable insights for this field of research.

The current study builds on the work by Daeglau et al. (2021b) and aims to explore whether watching nature documentaries might impact ERD development during MI-NF training. In this follow-up study, nature documentaries were replaced with quiet rest as the post-MI condition. The term post-MI condition refers to the activity performed immediately after completing an entire MI block. Quiet rest was chosen to minimize the confrontation with further sensory and auditory stimuli after MI-NF training and to reduce the associated cognitive load. We collected new data from a *quiet rest* group and compared it to the previously recorded data from the no-interference group (here called *documentaries* group) of the original study, to investigate any potential differences in ERD development.

## 2 Methods

### 2.1 Participants

Data were collected from 20 healthy participants (9 female, 18–35 years, M ± SD: 24.9 ± 4.2 years), all right-handed with normal or corrected vision, and no neurological or psychiatric conditions. Participants had not taken part in previous MI-NF studies and provided written informed consent. Handedness was assessed using the Edinburgh Handedness Inventory (Oldfield, 1971). To evaluate participants’ ability to engage in visual and kinesthetic MI, we used the short version of the Kinesthetic and Visual Imagery Questionnaire (KVIQ-10), which measures the vividness of both imagery modalities separately (Malouin et al., 2007). Three participants were excluded for not meeting inclusion criteria (two for signs of depression; one for ambiguous handedness), leaving data from 17 participants (8 female, 18–35 years, M ± SD: 25.2 ± 4.2 years) for analysis. These data was compared to 17 previously collected datasets (10 female, 23–32 years, M ± SD: 25.8 ± 2.5 years) (Daeglau et al., 2021b).

### 2.2 Study design

**Figure 1:**
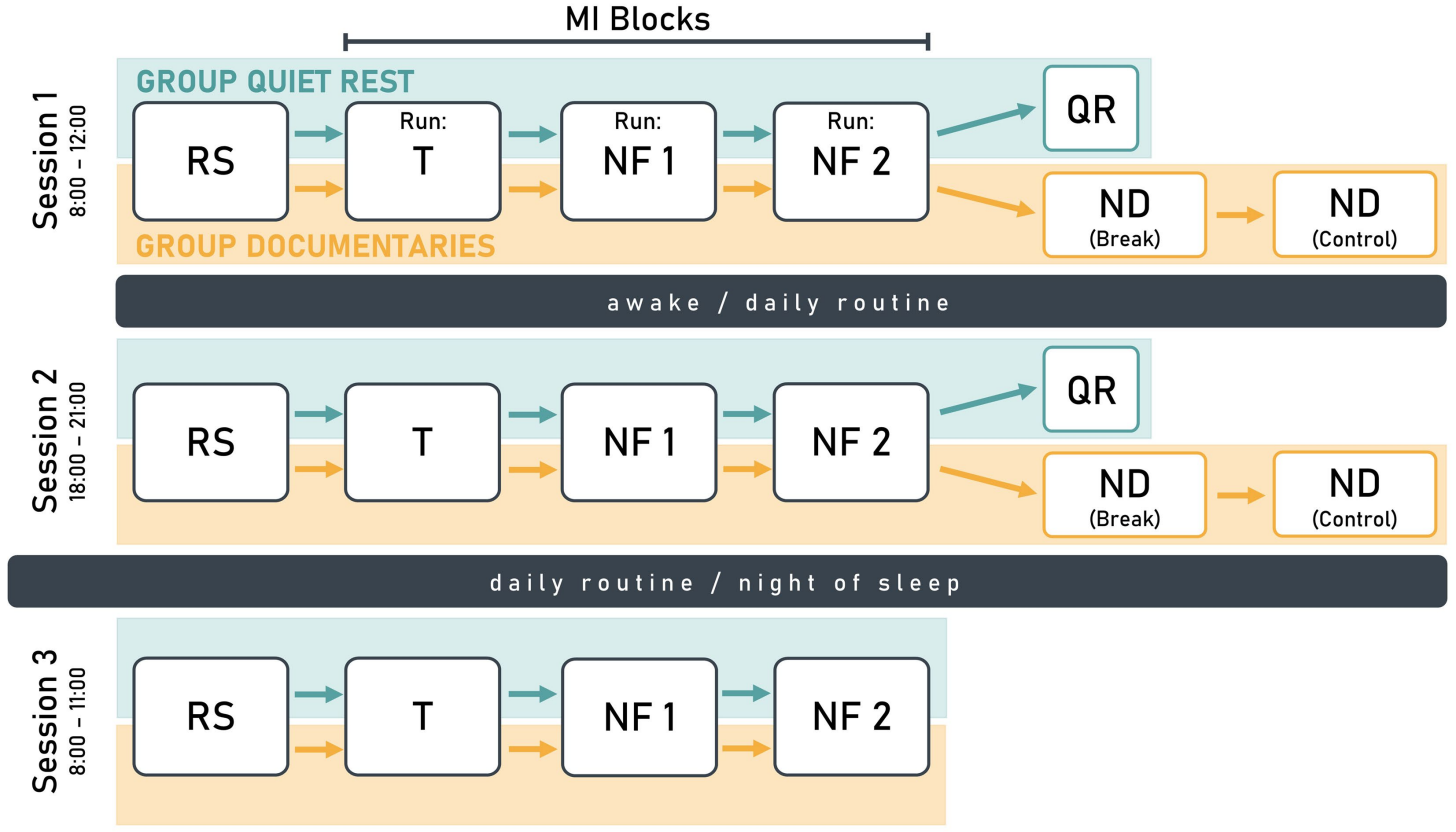
Schematic illustration of the study design visualizing the similarities and differences between the study groups *quiet rest* (turquoise) and *documentaries* (orange). Each participant completed three sessions with session 1 and 2 on the same day, and session 3 on the following day. The design included a resting-state measurement (RS), a training run (T), and two neurofeedback runs (NF). The *quiet rest* group had a 10-min quiet rest period (QR) after NF2, while the *documentaries* group watched a nature documentary (ND) during a 30-min break and a subsequent 30-min control condition.

Each participant completed three sessions: One in the morning, one in the evening on the same day, and one on the following morning (Figure 1). In the first session, participants practiced a sequential finger-tapping task for MI, moving each finger to touch their thumb at a rate of 1 Hz with both hands. Each session started with a 2-min resting-state measurement with eyes open, followed by an MI block consisting of three runs. During MI, participants were asked to kinesthetically imagine the finger-tapping task from a first-person perspective, focusing on the sensations they would feel if they were physically performing the task.

In the first run of the MI block, participants mentally rehearsed the finger-tapping task without receiving NF. The EEG data collected in the first run were used to train the NF parameters for the second run and is therefore referred to as training run (T). In the following two NF runs (NF1 and NF2), MI was supported by real-time NF, which visualized individual ERD strength.

For the *quiet rest* group, NF2 was followed by a 10-min quiet rest period, during which participants relaxed and focused on a fixation point on the screen. For the *documentaries* group, the overall session structure was identical to that of the *quiet rest* group, except for the post-MI condition. Here, NF2 was followed by two 30-min blocks, during which participants passively watched a nature documentary. The quiet rest period was designed to provide a controlled post-MI resting phase, aligning with motor learning research suggesting that short post-training waking rest supports skill consolidation (Hotermans et al., 2006; Brokaw et al., 2016; Humiston and Wamsley, 2018). Extending this period to 60 min, as in the *documentaries* group, was deemed impractical due to the potential for boredom and disengagement.

The second session of the study replicated the structure of the first session. In the third session, participants only completed a 2-min resting-state measurement and one MI block (three runs: T, NF1, NF2). Participants were instructed to maintain their regular daily routines between sessions, avoiding exhausting exercise and naps.

### 2.3 Experimental procedure

**Figure 2:**
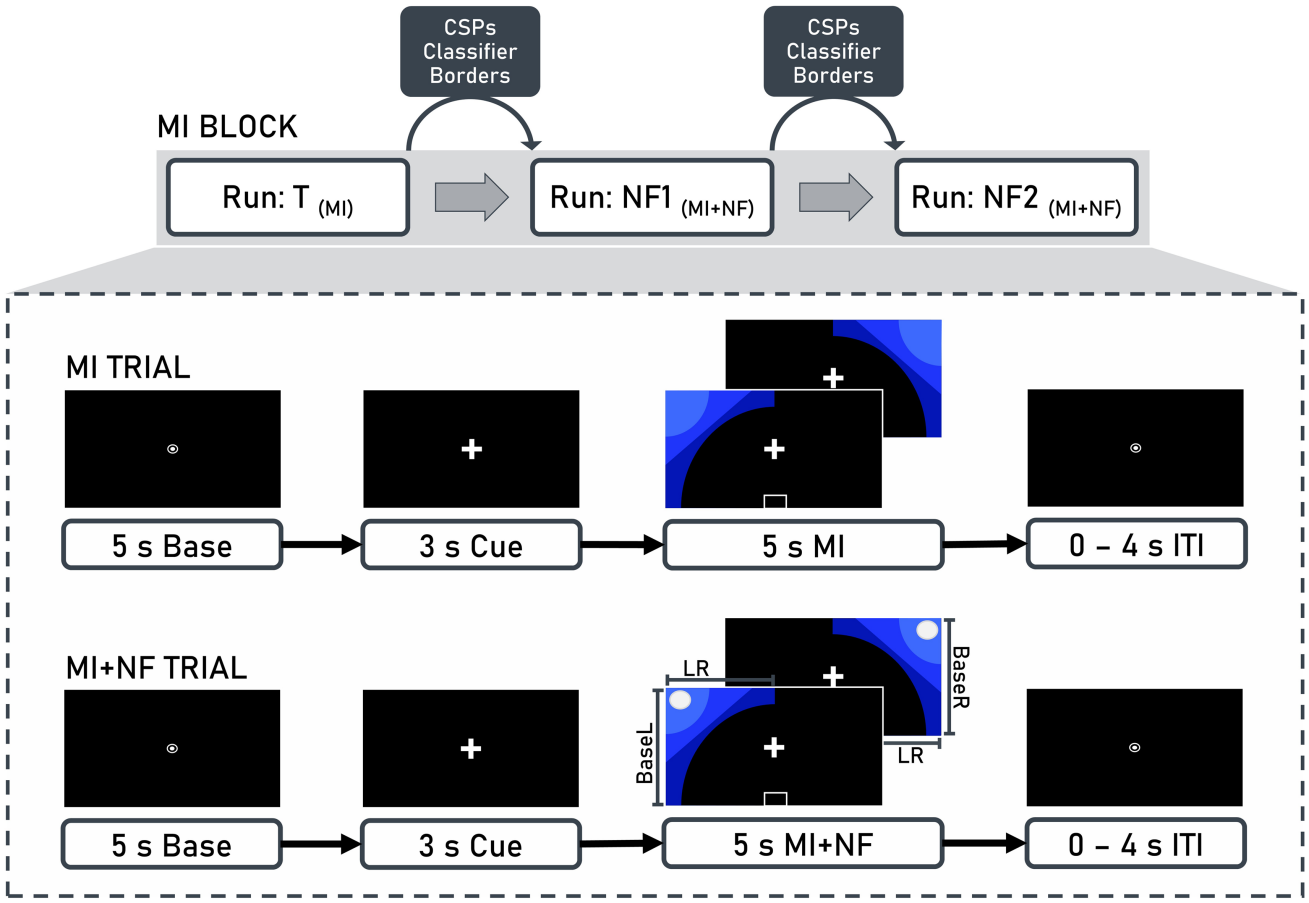
Structure of motor imagery (MI) blocks and trials. Each block consisted of three runs: Training (T), neurofeedback 1 (NF1), and neurofeedback 2 (NF2). Between runs, common spatial patterns (CSP), classifiers (BaseL, BaseR, LR), and borders were calculated based on the EEG data recorded from the previous run. These values were then used for the presentation of neurofeedback in the subsequent run. A trial included a 5-s baseline, a 3-s cue, and a 5-s motor imagery interval, which could either be without (MI trial) or with (MI + NF trial) neurofeedback, followed by an inter-trial interval (ITI) ranging from 0 to 4 s.

The experiment was controlled using OpenViBE 0.17.1 (Renard et al., 2010), which was also used in the original study by Daeglau et al. (2021b). The procedure was identical for both the *quiet rest* and *documentaries* groups. Each 10-min run consisted of 40 trials (20 left-hand trials, 20 right-hand trials) in pseudorandomized order (Figure 2). Each trial began with a 5-s baseline period, followed by a 3-s preparation cue. During the 5-s MI interval, blue graphics appeared on either the left or right side, indicating which hand should be used for the MI of the finger-tapping task. Trials were separated by an inter-trial interval ranging from 0 to 4 s.

When feedback was provided, a white ball moved along the *x*-axis (representing ERD lateralization) and the *y*-axis (representing contralateral ERD strength) based on two linear discriminant analysis classification results. Participants used kinesthetic MI of the finger-tapping task to steer the ball to the upper left corner for left-hand trials and the upper right corner for right-hand trials. They were instructed to focus on the fixation symbols, remain relaxed, and minimize physical movement.

### 2.4 Group settings and procedures

Settings and procedures for group *quiet rest* were matched as closely as possible to those of group *documentaries* in all aspects except for the post-MI condition. Both groups followed the same experimental procedure, including inclusion criteria, identical laboratory environment, pre-experiment assessments (Edinburgh Handedness Inventory, KVIQ-10), MI task and structure of MI blocks. A standardized instruction sheet was used to ensure consistency in explaining the experimental procedure and visualizing the task for participants. The sessions for both groups were scheduled at the same times of day, and the overall session structure was kept as identical as possible. The only systematic difference between groups was the post-MI condition and, consequently, the session duration. Additionally, data collection for the two groups was conducted by different female experimenters.

### 2.5 Data acquisition

EEG and surface electromyography data were recorded using a BrainAmp Amplifier System (Brain Products GmbH, Gilching, Germany). EEG settings included a 0.1 μV amplitude resolution, 500 Hz sampling rate, and 0.016–250 Hz online analogue filter. Impedance was reduced to below 10 kΩ. EEG data were collected from 64 sintered Ag/AgCl electrodes arranged in an equidistant layout on a customized, infracerebral electrode cap (EasyCap, Herrsching, Germany; caps that include electrode positions below the cerebrum), with a central frontopolar electrode as ground, a nose-tip reference, and two electrodes below the eyes to capture eye movements.

Electromyography data, measuring muscle activity of both hands, had impedance reduced to below 100 kΩ. Electrodes were placed over the muscle belly and the proximal base of the Flexor digitorum superficialis and the Abductor pollicis longus, with clavicle electrodes serving as reference and ground. Data acquisition during MI was managed with OpenViBE Server 0.17.1, while BrainVision Recorder (version 1.20.0506) was used for resting-state recordings.

### 2.6 Data analysis

Online and offline processing of the EEG and electromyography data followed the same protocol and utilized the same software as in Daeglau et al. (2021b), ensuring methodological consistency and allowing for a direct comparison between the newly recorded dataset and the existing one. The primary distinction between the two groups was the altered post-MI condition.

#### 2.6.1 Online processing

Within each MI block, raw EEG data were preprocessed online for real-time NF based on ERD over sensorimotor areas in the mu (8–12 Hz) and beta (13–30 Hz) bands during MI. Between MI runs, Common Spatial Patterns (CSP) analysis, classifier training, and border computation were conducted, using the results as baseline and threshold values for the NF in subsequent runs. Parameters from the training run calibrated NF1, while NF1 data calibrated NF2 (Figure 2).

EEG data from 49 central channels were filtered in the mu and beta bands using high-pass (8 Hz, order: 826) and low-pass (30 Hz, order: 220) FIR filters. Data were segmented into epochs from 0.5 to 4.5 s after MI onset, categorized by left-and right-hand movements, and epochs containing artifacts were rejected. CSP analysis was performed using the EEGLAB toolbox (version 14.1.1.) (Delorme and Makeig, 2004) in MATLAB (version 9.3; MathWorks, Natick, Massachusetts, USA, RID:SCR_001622). This common approach minimizes the impact of volume conduction and allows for an optimized extraction of movement-related neural patterns. Given the importance of precise feature extraction for MI-NF applications, MATLAB’s implementation provided greater control over preprocessing steps and feature selection than OpenViBE’s native implementation. A CSP analysis pipeline (Ramoser et al., 2000) identified spatial brain patterns correlating with left-and right-hand MI by applying spatial filtering that weights electrode contributions based on their classification relevance, enhancing feature extraction and classification. The two most neurophysiologically plausible CSPs for each run (one for left-hand and one for right-hand trials) were manually selected based on their ability to maximize the variance between movement classes while preserving physiologically meaningful patterns over sensorimotor areas. The selected filter coefficients were then used for classifier training in OpenViBE.

For the classifier training and border calculation in OpenViBE, raw EEG data were spatially filtered using the CSP coefficients and temporally filtered with a 4th-order Butterworth filter (8–30 Hz, 0.5 dB pass band ripple). Data were epoched from 0.5 to 4.5 s after MI onset for MI activity and from −7 to −3 s before MI onset for baseline activity. The data were divided into 1-s bins with a 0.9375-s overlap and logarithmic power of these bins served as features for linear discriminant analysis with seven-fold cross-validation (Fisher, 1936). Three classifiers controlled the NF ball’s movement: Horizontal movement (classifier LR) was controlled by the degree of lateralization, the difference between left-and right-hand MI. Vertical movement (classifier BaseL and BaseR) was controlled by the difference of contralateral activity during MI compared to baseline activity for left-hand (BaseL) and right-hand (BaseR) trials. Border values, defining the display range for on-screen NF, were calculated from cross-validation results, representing the upper quartiles of the three classifiers.

#### 2.6.2 Offline processing

Electromyography data were filtered (high pass FIR, cut-off 25 Hz, hamming window, order: 264), noise-cleaned using a wavelet signal denoiser toolbox (Daubechies 4 wavelet), and epoched from −9 to 7 s relative to MI onset. Trials with movement artifacts were excluded if the 250-sample centered moving standard deviation (MSD) exceeded the standard deviation by 2.5 times.

Independent component analysis was performed for EEG artifact rejection (Delorme and Makeig, 2004). Bad channels were identified using the EEGLAB extension trimOutlier ^1^with an upper and lower boundary of two standard deviations from the mean standard deviation across all channels and were excluded from further analysis (M ± SD of identified channels: 1.37 ± 0.63, range: 0–3 channels). A copy of the dataset was low-pass filtered (FIR, 40 Hz, hamming window, filter order: 166), down-sampled to 250 Hz and high-pass filtered (FIR, 1 Hz, hamming window, order: 414). Data were epoched in 1-s segments, and epochs containing artifacts rejected. The unmixing matrix derived from this process was applied to the original, unfiltered EEG dataset to identify and remove components associated with artifacts. ICLabel (Pion-Tonachini et al., 2019) and the Eye-Catch approach (Bigdely-Shamlo et al., 2013) were used to detect components associated with eye, muscle, and heart activity. Components were then visually inspected, and those identified as artifacts were excluded from further analysis.

The corrected data were filtered (low-pass FIR filter, cut-off frequency 30 Hz, hamming window, order: 220, Fs = 500 Hz; high-pass FIR filter, cut-off frequency 8 Hz, hamming window, order: 826, Fs = 500 Hz), re-referenced to common average, and interpolated for bad channels. Data were then segmented separately for left-and right-hand trials from −7 to 9 s relative to MI onset, and baseline correction was performed from −6 to −4 s. As in online processing, data were then reduced to the central 49 channels and CSP analysis was performed. In contrast to online processing, CSP were not calculated for each run individually but for the three MI runs per MI block together. The two most neurophysiologically plausible filters, one for left-hand and one for right-hand MI, were selected. For contralateral activity, we applied each hand’s CSP filter to its respective EEG data: right-hand filter to right-hand EEG and left-hand filter to left-hand EEG. For ipsilateral activity, we applied the right-hand filter to left-hand EEG and the left-hand filter to right-hand EEG.

Following this step, ERD extraction was conducted according to the method described by Pfurtscheller and Lopes da Silva (1999). Brain activity was averaged across all trials in a run within the MI interval for each block and participant. Relative contralateral ERD from left-and right-hand trials was averaged. ERD was also averaged over both NF runs (NF1 and NF2) within the MI interval for further statistical analysis. To ensure that pre-existing variations in baseline power did not account for potential differences in MI-NF-ERD between groups, we conducted a baseline power comparison. Baseline power was defined as the average EEG activity within the -6 s to -4 s interval before MI onset. For each participant, baseline power values were averaged within an MI block across all trials for both NF runs (NF1 and NF2) to obtain a single baseline measure per MI block. The baseline power values were then compared between the *quiet rest* and *documentaries* groups.

### 2.7 Statistical analyses

Paired *t*-tests were used to compare training run ERDs (T-MI-ERD) with averaged NF run ERDs (NF-MI-ERD) to assess the impact of NF on MI-ERD, under the assumption that T-MI-ERD would be less negative than NF-MI-ERD (measure 1 > measure 2). A repeated measures ANOVA with block as the repeated measures factor and NF-MI-ERD as level was used to evaluate across-block gains in NF-MI-ERD. To compare NF-MI-ERD between the *quiet rest* and *documentaries* groups, another repeated measures ANOVA was conducted with blocks as repeated measures factor, NF-MI-ERD as level, and group as a between-subjects factor. Mauchly’s test (Mauchly, 1940) was used to assess sphericity, and Greenhouse–Geisser corrections were applied when necessary. Interaction effects were investigated with independent *t*-tests comparing NF-MI-ERDs between groups within each block. Effect sizes were reported as eta-squared (*η*^2^) for ANOVAs (90% CI) and Cohen’s d (*d*) for *t*-tests (95% CI) (Steiger, 2004). The Bonferroni-Holm method was applied to correct for multiple comparisons (Holm, 1979). Additionally, a baseline power analysis was conducted. The Shapiro–Wilk test was used to assess normality, and Levene’s test examined the equality of variances. Independent samples *t*-tests were performed to compare baseline power between the *quiet rest* and *documentaries* groups within the same MI block.

## 3 Results

### 3.1 Neurofeedback effect group quiet rest

The effects of NF on MI-ERD within each block were assessed using paired *t*-tests, comparing the means of NF-MI-ERD with those of T-MI-ERD (Table 1). The results revealed a significantly stronger expression of ERD in all three blocks when MI was supported by NF (*df* = 16, *p* < 0.001, *d* > 0.9 for each block), indicating that NF reliably enhanced ERD.

**Table 1:** Paired *t*-tests for comparison of training run ERD (T) and NF runs ERD (NF).

block	*t*	*df*	*p*	*p_holm_*	*d*	95% CI	*N*	M_T_	SD_T_	M_NF_	SD_NF_
1	5.185	16	<0.001	<0.001	1.258	(0.704, ∞)	17	−8.176	18.510	−29.285	9.589
2	4.041	16	<0.001	<0.001	0.980	(0.479, ∞)	17	−14.168	8.428	−25.939	11.003
3	3.932	16	<0.001	<0.001	0.954	(0.457, ∞)	17	−8.087	16.656	−24.731	10.582

**Figure 3:**
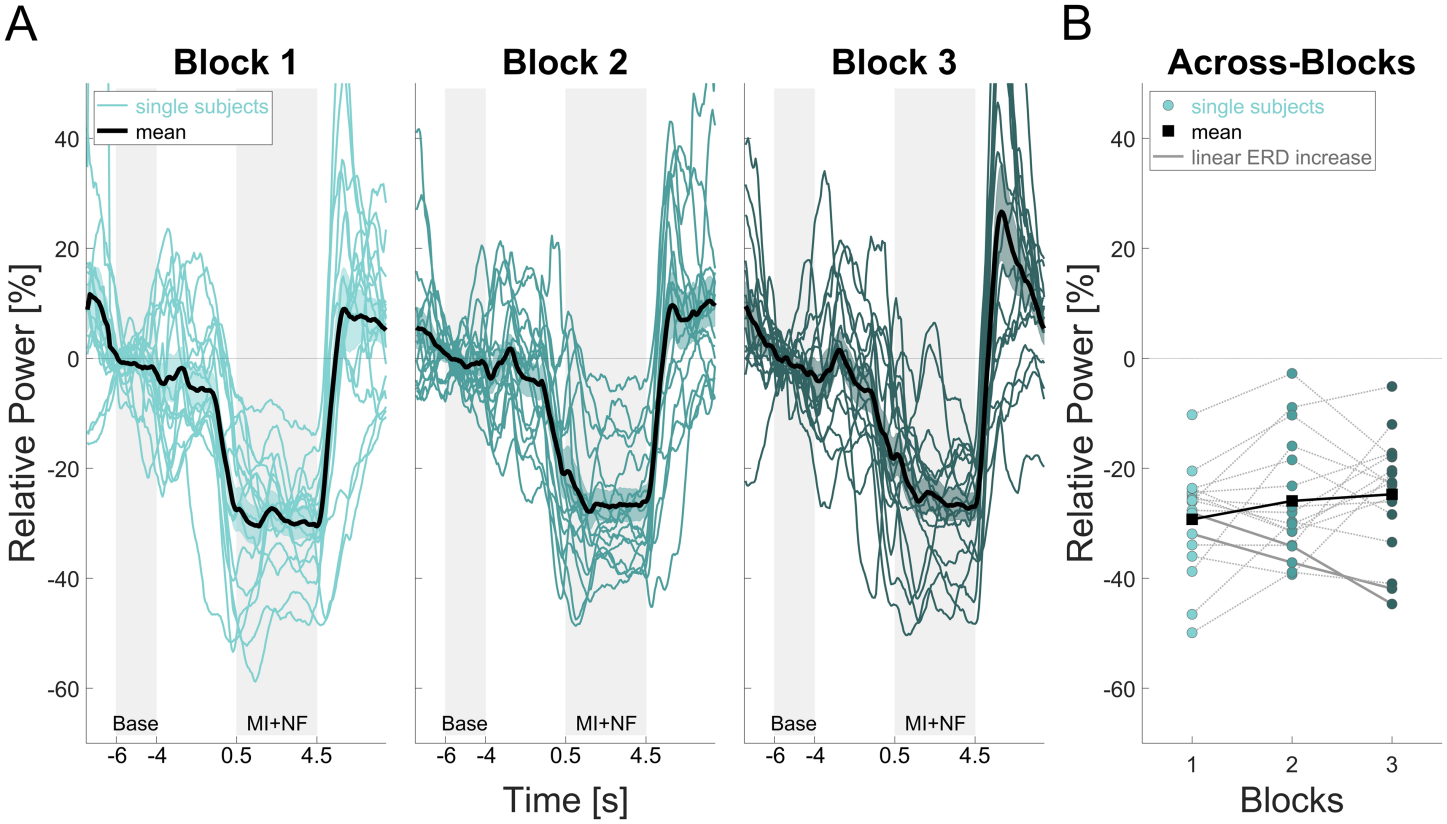
NF-MI-ERD time courses for the *quiet rest* group. The *y*-axis represents relative power [%], while the *x*-axis indicates time [s] **(A)** and block **(B)**. Panel **(A)** displays individual contralateral NF-MI-ERD time courses for each participant across all blocks, along with the calculated average time course for all participants within each block. Panel **(B)** illustrates the mean contralateral NF-MI-ERD values for each participant (a single value per block) and the overall averaged NF-MI-ERD across all participants in each block. The connecting lines highlight changes in NF-MI-ERD means for both individual participants and the group across the three blocks.

The NF-MI-ERD time courses for all participants across each block are illustrated in Figure 3. The characteristic desynchronization of brain activity during MI was observed consistently across all participants and blocks (Figure 3A).

To further assess the development of NF-related ERD gains across blocks, a repeated measures ANOVA was conducted. The analysis showed no statistically significant changes in NF-MI-ERD over the three blocks (*F*_(2,32)_ = 1.735, *p* = 0.193, *η*^2^ = 0.098). Notably, NF-MI-ERDs were highly variable, with only two participants showing a linear increase in ERD over time, as highlighted by the solid grey lines in Figure 3B.

### 3.2 Comparison of groups quiet rest and documentaries

**Figure 4:**
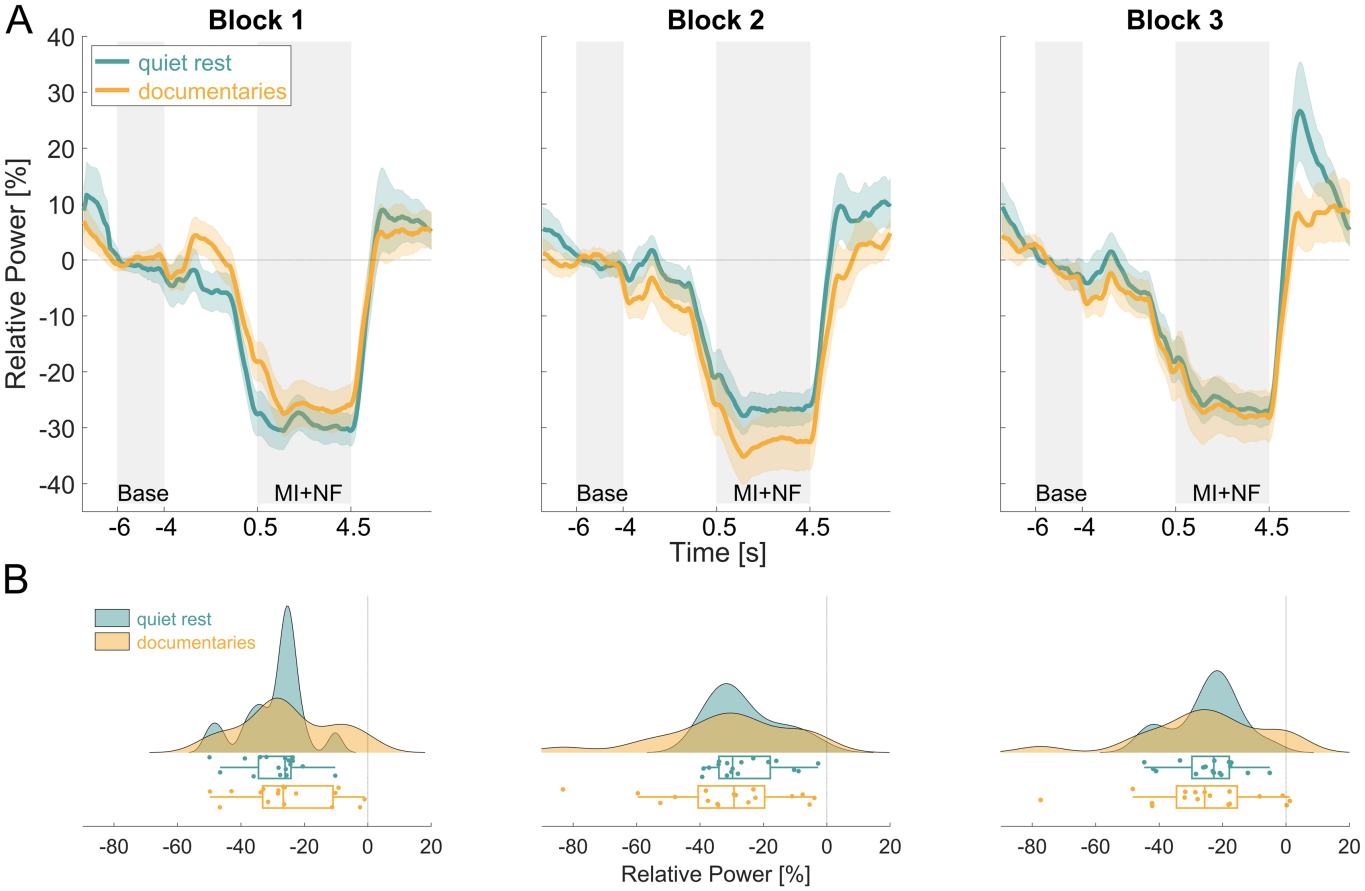
Comparison of NF-MI-ERD results between the *quiet rest* (turquoise) and *documentaries* (orange) groups. Panel **(A)** displays the averaged contralateral NF-MI-ERD time courses for both groups across all three blocks. The *y*-axis represents relative power [%], while the *x*-axis indicates time [s]. Panel **(B)** illustrates the distribution of single-subject mean contralateral NF-MI-ERD values for both groups across each block, with the *x*-axis representing relative power [%].

The time courses of NF-MI-ERD for both the *quiet rest* and *documentaries* groups, along with the distribution of single-subject mean NF-MI-ERD values are illustrated in Figure 4. ERD was evident in both groups throughout the experiment, with the most pronounced differences occurring in block 2 (Figure 4A). However, these differences were subtle and did not display a clear trend across the blocks. The raincloud plots reveal that the distribution of mean NF-MI-ERD values is broader in the *documentaries* group compared to the *quiet rest* group, indicating greater variability in the ERD response across all blocks (Figure 4B).

A baseline power comparison was conducted to test for potential differences between groups. Prior to this, the Shapiro–Wilk test indicated that baseline power values were normally distributed in both groups across all blocks (all *p* > 0.05) and Levene’s test confirmed that variance was homogenous between groups (all *p* > 0.05). No significant differences in baseline power were found within the same MI blocks (Block 1: *df* = 32, *p* = 0.28 *d* = −0.377; Block 2: *df* = 32, *p* = 0.106, *d* = −0.571; Block 3: *df* = 32, *p* = 0.321, *d* = 0.346). These results confirm that baseline EEG activity was comparable between groups.

To assess the effect of NF across blocks and compare the two groups, a repeated measures ANOVA was conducted. The analysis showed no statistically significant main effect of NF across blocks (*F*_(2,64)_ = 1.831, *p* = 0.169, *η*^2^ = 0.010), nor was there a significant difference between groups (*F*_(1,32)_ = 0.055, *p* = 0.815, *η*^2^ = 0.001). However, a significant interaction effect between block and group was observed, albeit with a small effect size (F_(2,64)_ = 3.526, *p* = 0.035, *η*^2^ = 0.019). This interaction was further investigated by post-hoc independent *t*-tests to determine if the NF-MI-ERDs differed between groups within each block (Table 2). Bonferroni-Holm corrections for multiple comparisons were applied. No significant differences were found between the *quiet rest* and *documentaries* groups in any of the individual blocks (Block 1: *df* = 32, *p* = 0.7, *d* = 0.325; Block 2: *df* = 32, *p* = 0.885, *d* = −0.365; Block 3: *df* = 32, *p* = 0.822, *d* = −0.078).

**Table 2:** Independent *t*-tests for comparison of blocks between the *quiet rest* (QR) and the *documentaries* (D) group.

block	*t*	*df*	*p*	*p_holm_*	*d*	95% CI	*N*	M_QR_	SD_QR_	M_D_	SD_D_
1	0.948	32	0.350	0.700	0.325	(−4.588, 12.580)	17	−29.285	9.589	−25.289	14.490
2	−1.064	32	0.295	0.885	−0.365	(−17.679, 5.548)	17	−25.939	11.003	−32.005	20.773
3	−0.226	32	0.822	0.822	−0.078	(−12.349, 9.878)	17	−24.731	10.582	−25.966	19.851

## 4 Discussion

This study investigated the development of ERD induced by MI-NF training across three blocks. Specifically, it aimed to assess how ERD evolves with repeated MI-NF training and whether the post-MI condition influences this process. To explore these potential contextual effects, we collected new data from participants who engaged in MI-NF training followed by quiet rest (group *quiet rest*). This dataset was compared with previously recorded data from Daeglau et al. (2021b), in which participants watched nature documentaries after MI blocks (group *documentaries*) as both a control and break condition. The comparison sought to determine whether the post-MI condition - nature documentaries versus quiet rest - impacts the development of NF-related ERD across blocks.

We found that NF significantly increased ERD in all three blocks for the *quiet rest* group compared to training runs without NF. We investigated whether this enhancement strengthened over blocks to indicate an across-block NF gain, but found no significant difference, which is consistent with Daeglau et al. (2021b). A direct comparison between the *quiet rest* and *documentaries* groups revealed no significant differences in baseline power and NF-MI-ERD (Figure 4A). Both groups demonstrated similar overall trends in ERD, with slight variation in the progression of ERD across blocks between groups. While a block-by-group interaction effect suggests some variability in ERD patterns, post-hoc analyses could not confirm these differences. The small effect size (*η*^2^ = 0.02) implies that the variation between blocks may not represent a practically meaningful difference in ERD progression between the two groups. This suggests that ERD expression remained stable over time, regardless of whether participants engaged in quiet rest or watched nature documentaries as a post-MI condition. This indicates that altering the post-MI condition did not affect the evolution of NF-MI-ERD, suggesting that watching nature documentaries was not responsible for the absence of an across-block NF gain in the original study.

Similar studies with different methodologies have demonstrated stronger contralateral ERD (Ono et al., 2013; McWhinney et al., 2018; Foldes et al., 2020) and improved brain activity control (Wolpaw and McFarland, 2004; McFarland et al., 2010) in the course of MI-NF training. The question remains why no gain in ERD was observed in both the original study and our newly collected dataset.

### 4.1 Simplicity of the finger-tapping task

One possible explanation for the observed results is the simplicity of the finger-tapping task, which was chosen as the movement sequence for MI. Participants were instructed to imagine their thumb sequentially touching the index, middle, ring, and little finger at a rate of one touch per second. Importantly, the task did not require participants to memorize a complex sequence of finger movements. Before the first MI block, the task was demonstrated and physically practiced until participants felt comfortable with the sequence and the required pace of 1 Hz. Due to the simplicity of the task, participants learned it quickly. Additionally, during the KVIQ assessment, all participants reported an understanding of the difference between visual and kinesthetic MI and indicated being capable of applying kinesthetic MI. Given this, along with the presence of a typical ERD across all participants in all MI blocks, particularly in the first MI block (see Figure 3A), we are confident that participants were able to perform the kinesthetic MI of the finger tapping task effectively.

Research shows that complex or varied movements can enhance motor performance and learning after MI training (Allami et al., 2008; Rozand et al., 2016; Ruffino et al., 2017). The finger-tapping task, used in various MI-NF studies (Korman et al., 2007; Debarnot et al., 2009; Zich et al., 2015), may have allowed participants to quickly transition into the automatic phase of motor learning. This would reduce cognitive load, potentially diminishing the increase in ERD. As Pfurtscheller and Lopes da Silva (1999) reported, ERD is strongest when a movement sequence is newly learned but decreases once the movement becomes automatic. Similarly, Wolpaw and McFarland (2004) showed that brain-computer interface users initially rely on MI to generate control-relevant EEG activity, but as their performance improves, control becomes more automatic. Although their study involved a larger training paradigm with more sessions and trials, the underlying principle, that learned motor tasks require less cognitive effort and exhibit reduced ERD, might also apply for the present study. This could explain the slight trend towards a less strong ERD observed when averaging NF-MI-ERD across participants in the *quiet rest* group (Figure 3B).

### 4.2 Neurofeedback visualization

Additionally, the type of NF used could also have impacted ERD development. Studies have shown that more realistic and multidimensional NF approaches can promote stronger ERD (Ono et al., 2013; Liang et al., 2016; Braun et al., 2016; McWhinney et al., 2018) compared to two-dimensional, object-based NF. For instance, Ono et al. (2013) demonstrated that a three-dimensional anatomically congruent NF elicited greater ERD than conventional bar-based NF. Similarly, Liang et al. (2016) found that adding multimodal sensory feedback, such as vibrotactile stimulation, enhanced MI-based NF performance. Braun et al. (2016) showed that virtual reality NF improved motor-related brain activity more effectively than traditional two-dimensional feedback, while McWhinney et al. (2018) reported that task engagement was higher when NF provided richer and more interactive cues. Therefore, NF methods that incorporate more complex, dynamic feedback or that engage multiple sensory modalities may provide richer information and thereby enhance the brain’s ability to modulate ERD more effectively. The relatively simplistic NF design used in this study, with limited feedback dimensionality, might have constrained the participants’ ability to fully engage with the task and optimize ERD. Incorporating more challenging MI tasks alongside a more sophisticated, multidimensional NF approach could potentially improve the development of contralateral ERD across blocks, leading to more pronounced changes in brain activity over time.

### 4.3 ERD variability

Another important consideration is the pronounced inter-and intra-individual variability in ERD, a well-known phenomenon in MI research (Ahn and Jun, 2015; Wriessnegger et al., 2020; Leeuwis et al., 2021), that could affect MI/MI-NF outcomes. Such differences may arise from context factors within and beyond the experimental task, including character traits, imagery strategies and capability, task engagement, time-variant cognitive factors, brain structure, and session structure (Rulleau et al., 2015; Jeunet et al., 2015; Ahn and Jun, 2015; Rulleau et al., 2018; McWhinney et al., 2018; Lee et al., 2019; Saha and Baumert, 2019; Daeglau et al., 2021a; Leeuwis et al., 2021).

Given this variability in ERD responses, we applied CSP filtering as a preprocessing step to enhance the spatial specificity of MI-related neural activity by maximizing the discriminability of oscillatory patterns associated with MI. This is a common approach in MI-NF studies (Ramoser et al., 2000) because it optimally extracts the most relevant task-related EEG components, reducing volume conduction effects and improving the signal-to-noise ratio compared to single-channel analyses.

In this study, all participants in the *quiet rest* group exhibited detectable NF-MI-ERD in each block, yet substantial variability was evident across individuals, although CSP filtering was employed. When analyzing averaged values across blocks, the progression of ERD was notably inconsistent. Some participants showed a reduction in ERD from Block 1 to Block 2, while others displayed the opposite trend, with an increase in ERD. Similarly, transitions from Block 2 to Block 3 reflected comparable variability. Only two participants demonstrated the expected pattern, where NF-MI-ERD became progressively more negative from Block 1 to Block 3, aligning with the anticipated NF-gain. A similar pattern was observed in the *documentaries* group, where variability in ERD expression was even more pronounced (see Figure 4B). In particular, Block 2 and Block 3 revealed substantial differences between participants, with some exhibiting strong ERD while others showed markedly weaker responses.

This pronounced inter-and intra-individual variability underscores the complexity of achieving consistent ERD progression during MI-NF training and highlights the necessity of individualized approaches to optimize training outcomes.

### 4.4 Limitations

While this study provides valuable insights into ERD development during MI-NF training, there are several factors to consider for future research. The sample size, though adequate for initial analysis, could be expanded to capture more subtle effects. One notable difference between the two groups was the length of the post-MI condition. The MI blocks were identical in both groups, but the *documentaries* group had a longer post-MI session, with 30 min of passively watching nature documentaries used as both a break and control condition, whereas the *quiet rest* group had a 10-min resting-state measurement. Despite the shorter session length after the MI blocks in the *quiet rest* group, our results showed no significant differences in ERD development between the two groups. This suggests that the length of the session following MI-NF may not have a strong impact on ERD outcomes in this context.

Furthermore, some limitations should be considered when comparing the two groups. From the study’s inception, we ensured that all procedures in the *quiet rest* group paralleled those of the *documentaries* group as closely as possible. Both groups were matched in terms of sample size, age, and gender distribution to minimize demographic biases. Additionally, the same NF protocol and analysis pipeline were applied to both groups to maintain consistency. However, some differences remain. Data collection was conducted by two different experimenters, but both were of the same gender, allowing us to rule out potential differences in gender-related experimenter effects (Roc et al., 2019). Additionally, there was a time gap between the data collection periods of the two groups, which could have introduced slight variations in the population from which participants were recruited or laboratory conditions. While every effort was made to standardize the experimental setup, these factors may have contributed to variability in the results and should be considered when interpreting the findings.

## 5 Conclusion

This study found no evidence that watching nature documentaries interfere with ERD development in MI-NF training, contrary to previous speculation. Replacing nature documentaries with quiet rest as the post-MI condition did not lead to the expected MI-NF training gain, which aligns with the findings of Daeglau et al. (2021b). However, several other unexplored contextual factors could influence NF-MI-ERD outcomes either positively or negatively (Daeglau et al., 2021a; Ladda et al., 2021). Future research should not only investigate these contextual factors but also consider employing more complex movement sequences to better capture the dynamics of NF-MI-ERD gains over time. Understanding the functional significance of these gains and their relationship to individual performance may provide valuable insights into optimizing MI-NF training protocols.

## Data Availability

The datasets generated and analyzed during this study are not publicly available but can be obtained from the corresponding author, JD, upon reasonable request. Requests to access the datasets should be directed to Jennifer Decker, jennifer.decker@uni-oldenburg.de.

## Footnotes

^1^ https://sccn.ucsd.edu/wiki/EEGLAB_Extensions
